# Application effects of information-based health management combined with knowledge-attitude-practice health education model in hypertensive patients

**DOI:** 10.1590/1414-431X2026e15576

**Published:** 2026-09-07

**Authors:** Jiangwen Duan, Qitao Jin

**Affiliations:** 1 Department of Health Education, Zhoushan Center for Disease Control and Prevention (Zhoushan Health Supervision Institute), Zhoushan, Zhejiang, China

**Keywords:** Information-based health management, Knowledge-attitude-practice health education model, Hypertension, Adherence, Quality of life

## Abstract

This study investigated the application effects of information-based health management combined with the knowledge-attitude-practice (KAP) health education model in hypertensive patients. Two hundred hypertensive patients were randomized into a control group or an intervention group. Patients in the control group received conventional health management and health education, whereas those in the intervention group received information-based health management plus the KAP health education model. The blood pressure control rate, pre- and post-management systolic and diastolic blood pressures, medication adherence, self-management abilities [assessed using the Hypertension Patient Self-Management Behavior Rating Scale (HPSMBRS)], health behaviors [examined using the Health-Promoting Lifestyle Profile II (HPLP-II)], quality of life [estimated using the Generic Quality of Life Inventory-74 (GQOLI-74)], and patient satisfaction with management were compared between the two groups. Following management, the blood pressure control rate in the intervention group was higher relative to the control group (P<0.05). Compared to pre-management levels, both groups showed noticeable reductions in systolic and diastolic blood pressures post-management, with lower values observed in the intervention group (P<0.05). Both groups exhibited increased medication adherence, self-management abilities, health behavior scores, and quality of life, with higher values in the intervention group (P<0.05). Patient satisfaction with management was higher in the intervention group (P<0.05). Information-based health management plus the KAP health education model effectively improved blood pressure control, medication adherence, self-management ability, health behaviors, and quality of life in patients with hypertension, with high patient satisfaction.

## Introduction

Hypertension is a leading risk factor for cardiovascular morbidity and mortality and represents a persistent global public health challenge (1). Inadequate blood pressure control is associated with serious adverse outcomes, including renal impairment and the progression of existing cardiovascular and renal diseases (2). Despite the growing burden of hypertension, rates of disease awareness, treatment initiation, and effective blood pressure control remain consistently low worldwide (3). Evidence increasingly suggests that timely intervention and effective long-term management can substantially reduce hypertension-related morbidity and mortality (4).

Information-based health management in hypertension refers to the use of digital and technology-supported tools (such as remote blood pressure monitoring, mobile health applications, telemonitoring, and patient education platforms) to assist patients in long-term disease self-management. Recent evidence suggests that digital health interventions can lead to greater reductions in blood pressure compared with standard care, particularly when remote monitoring and tailored strategies are incorporated (5). Furthermore, digital technology - such as home blood pressure monitoring systems, mobile applications, and wearable devices - has expanded opportunities to address barriers related to medication adherence and individualized care in hypertension management (6). In real-world settings, comprehensive digital self-management programs involving patient education and behavior change support have been shown to be effective for improving hypertension outcomes over time (7).

Given the central role of patient knowledge, attitudes, and behaviors in hypertension control, the knowledge-attitude-practice (KAP) model provides a useful theoretical framework for understanding and modifying health-related behaviors. In public health research, KAP-based assessments are commonly used to evaluate disease-related knowledge, risk perception, and behavioral practices (2). This approach is particularly valuable for exploring determinants of health behaviors and guiding targeted interventions (8,9). A previous study has highlighted the importance of hypertension-related knowledge, attitudes, and behaviors, as well as the critical role of dietary salt intake in both hypertension prevention and management (2).

Hypertension management continues to face substantial challenges worldwide, including large patient populations, regional and ethnic disparities, diverse dietary patterns, uneven distribution of healthcare resources, and insufficient public awareness of the disease. Many patients fail to monitor their blood pressure regularly, often due to limited understanding of hypertension-related risks or the perceived inconvenience of home blood pressure measurement. However, evidence regarding the combined application of information-based health management and the KAP health education model in hypertension remains limited. Against this background, the present study aimed to evaluate the effectiveness of integrating information-based health management with the KAP health education model in patients with hypertension.

## Material and Methods

### Ethics statement

Ethical approval for this research was granted by the Ethics Committee of Zhoushan Center for Disease Control and Prevention (Zhoushan Health Supervision Institute), with written informed consent obtained from all participants.

### General information

Two hundred hypertensive patients admitted from January 2022 to December 2024 were selected as the study subjects. Hypertension was diagnosed in accordance with the *2024 European Society of Cardiology (ESC) Guidelines for the Management of Elevated Blood Pressure and Hypertension* (10), defined as a systolic blood pressure (SBP) ≥140 mmHg and/or diastolic blood pressure (DBP) ≥90 mmHg documented on at least two separate clinical visits. Eligible participants were long-term local residents with adequate comprehension and communication abilities, allowing effective interaction with healthcare providers, accurate reporting of symptoms and treatment-related concerns, and reliable participation in follow-up assessments. Only patients with complete follow-up data were included. Patients were excluded if they had concomitant malignant tumors with an expected survival of less than 12 months, severe dysfunction of major organs, limb movement impairment, psychiatric disorders or cognitive impairment, or if they declined participation or withdrew from the study for personal reasons. No noticeable differences in general data were identified between the two groups (P>0.05), ensuring good comparability. Sample size estimation was performed based on published evidence (11) and pilot data. The expected mean SBP in the intervention group was 128.40 and 141.60 mmHg in the control group. Based on a two-sided α level of 0.05 and a power of 0.80, the sample size per group was calculated using the formula (Eq. 1) for two independent means (Eq. 2). After adjusting for a projected 20% loss to follow-up, the minimum required sample size was 38 participants per group. The final analysis included 50 patients in each group.

$$n=\frac{2\left(\sigma_{1}^{2}+\sigma_{1}^{2}\right){\left(Z_{\left(1-\alpha /2\right)}+Z_{\left(1-\beta \right)}\right)}^{2}}{{\left(\mu_{1}-\mu_{2}\right)}^{2}}$$ (Eq. 1)

$$n=\frac{2\left({\mathrm{13.04}}^{2}+12.96^{2}\right){\left(\mathrm{1.96}+\mathrm{0.84}\right)}^{2}}{{\left(128.40-141.60\right)}^{2}}\approx 31$$ (Eq. 2)

## Methods

Participants in the control group received routine health management combined with conventional health education. Nursing staff established individual electronic health records for each patient, documenting blood pressure measurements, medical history, medication regimens, and lifestyle habits. Follow-up data were updated regularly. Standardized medication counseling was provided, emphasizing strict adherence to prescribed antihypertensive therapy and discouraging any unauthorized dosage adjustments. Patients were provided with educational brochures on hypertension prevention and management. Dietary guidance focused on promoting a balanced diet, with particular emphasis on low-salt and low-fat intake, as well as strict smoking cessation and alcohol restriction. Face-to-face follow-up visits were conducted once monthly, during which blood pressure was measured and recorded. For patients whose blood pressure was inadequately controlled, the frequency of follow-up was increased to twice per month. The duration of routine management was three months.

The intervention group received information-based health management plus the KAP health education model for a period of three months. Information-based health management was implemented using the WeChat platform and included the following components: 1) WeChat group management: a WeChat group entitled ‘*Hypertension Health Education*' was established and administered by experienced community nurses. Patients and community physicians were invited to join the group. Group announcements were released in advance, specifying the schedule for educational content delivery and online interactions. Health education materials integrating text and images were disseminated every Monday and Friday to encourage review and patient inquiries. A 60-min online nurse-patient interaction session was conducted every Wednesday to address patient questions. Frequently raised questions were systematically recorded. Patients with recurrent or complex concerns were contacted individually through private WeChat messaging for personalized responses. 2) WeChat official account management: a dedicated WeChat official account was created and structured into four sections: outpatient appointment services, nurse-patient communication, health education management, and healthcare provider introduction. The health education management section delivered information related to blood pressure control, medication precautions, and standardized blood pressure measurement techniques. Through the outpatient appointment section, patients could schedule visits in advance and submit symptoms or questions, enabling healthcare providers to prepare targeted consultations. 3) WeChat-based supervision and monitoring: a supervision mechanism targeting medication adherence, dietary habits, and physical activity was established. Patients were provided with hypertension self-management logs and instructed to record daily information on medication use (including timing, frequency, administration method, and adverse reactions), diet (cooking methods and food types), and exercise (duration and frequency). Patients were required to submit photographs of their completed logs to the WeChat group every Monday. Group administrators reviewed these records to assess patients' self-management status, identify individuals with suboptimal adherence, and explore underlying reasons through voice calls. When necessary, home visits or face-to-face consultations at community health service centers were arranged to deliver individualized support.

KAP health education model: 1) Establishment of the KAP health education team: a multidisciplinary KAP health education nursing team was formed, consisting of one attending physician from the Department of Cardiovascular Medicine, one head nurse, and several nursing staff members. Prior to study initiation, team members received standardized training on the KAP health education model and underwent competency assessments. Individualized education plans were developed following comprehensive assessments of patients' knowledge of hypertension, dietary practices, physical activity, and blood pressure monitoring behaviors. 2) Knowledge education: clear educational objectives, content standards, and evaluation methods were defined. Health education was delivered through multiple formats, including face-to-face instruction, video presentations, and guideline-based educational materials. A departmental health education WeChat group was established, supplemented by telephone follow-up. Educational content covered fundamental hypertension knowledge, risk factors, and the impact of unhealthy lifestyle behaviors on blood pressure. Patients' existing health problems were evaluated, and targeted solutions were proposed. Each session lasted 20-30 min and was conducted once weekly. 3) Attitude formation: following three consecutive knowledge education sessions, patients' attitudes and beliefs regarding treatment adherence and self-management were assessed. Individual counseling was provided to analyze and address inappropriate beliefs or misconceptions related to hypertension. Comparative examples of regular versus irregular physical activity were discussed, and the consequences of unhealthy dietary and exercise behaviors were explained. Personalized diet and exercise plans were formulated based on patients' preferences and habitual behavior. 4) Behavior guidance: behavioral interventions focused on reinforcing psychological support, dietary management, physical activity, and self-monitoring practices. Patients were encouraged to maintain daily records of dietary intake and prescribed exercise. Emphasis was placed on accurate and sustained adherence to prescribed medications, correct blood pressure measurement techniques, adequate sleep, regular daily routines, and minimizing late-night activities.

### Efficacy evaluation criteria

Blood pressure outcomes were assessed by measuring SBP and DBP levels at baseline and after three months of intervention. Blood pressure control was defined as achieving SBP and DBP values below 140/90 mmHg at follow-up.

Medication use during the three-month intervention period was documented, and medication adherence was compared between the two groups. Adherence was evaluated using the General Medication Adherence Scale (GMAS) (12), which yields a total score ranging from 0 to 15 points, with higher scores reflecting poorer adherence. Based on the GMAS score, patients were classified as complete adherence (0 points), partial adherence (1-6 points), or non-adherence (7-11 points). Overall adherence was calculated as (number of complete adherence+ partial adherence cases) / total cases × 100%.

Self-management capacity was assessed at baseline and after three months using the Hypertension Patient Self-Management Behavior Rating Scale (HPSMBRS) (13). This instrument evaluates six domains, including dietary management (10-50 points), medication management (4-20 points), emotional management (7-35 points), exercise management (3-15 points), disease monitoring (4-20 points), and work-rest balance management (5-25 points). Higher scores indicated better self-management performance.

Health-related behaviors were evaluated pre- and post-intervention using the Health-Promoting Lifestyle Profile II (HPLP-II) (14). It involves four dimensions: health responsibility, physical exercise, nutrition, and stress management, each with nine items, scored from 1-4. Higher scores reflected more favorable health behaviors.

Quality of life was measured at baseline and at three months using the Generic Quality of Life Inventory-74 (GQOLI-74) (15). This questionnaire assesses social function, psychological function, physical function, and material living conditions, each scored from 0-100. Higher scores reflected a better perceived quality of life.

Nursing satisfaction was assessed after three months of intervention using a hospital-developed management satisfaction questionnaire. The survey evaluated patients' satisfaction with nursing services across multiple aspects, with total scores ranging from 0 to 5 points. Scores greater than 4 were classified as very satisfied, scores between 2 and 4 as satisfied, and scores below 2 as dissatisfied. The overall satisfaction rate was calculated as the proportion of patients reporting very satisfied or satisfied responses among all participants in each group.

### Statistical analysis

SPSS 25.0 and GraphPad Prism 10.0 were utilized for statistical processing. Prior to group comparisons, the distribution of continuous variables was estimated with the Shapiro-Wilk test. Data satisfying normality assumptions are reported as means±SD, whereas non-normally distributed variables were summarized as median with interquartile range (P25, P75). For intergroup comparisons, normally distributed continuous variables were analyzed with the independent-samples *t*-test, while non-normally distributed variables were tested with the Mann-Whitney U test. Intragroup changes pre- and post-intervention were estimated by the paired-samples *t*-test for normally distributed data and the Wilcoxon signed-rank test for non-normally distributed data. Group differences in categorical variables were evaluated by calculating proportions and applying the chi-squared (χ^2^) test. A P value <0.05 indicated statistical significance.

## Results

### General information

Baseline characteristics, including gender ratio, age, duration of disease, educational background, and hypertension classification, were well balanced between the two groups, with no differences observed (P>0.05; Table 1).

**Table 1:** Comparison of general information between the two groups.

Indicator	Intervention group (n=100)	Control group (n=100)	χ^2^/*t*	P
Gender				
Male	55 (55%)	56 (56%)	0.020	0.887
Female	45 (45%)	44 (44%)		
Age (years)	64.24±5.80	65.59±6.63	1.532	0.127
Duration of disease (years)	9.97±2.05	10.31±2.07	1.186	0.237
Education background				
High school and below	65 (65%)	67 (67%)	0.089	0.7653
College degree or above	35 (35%)	33 (33%)		
Hypertension classification				
Class I	22 (20%)	24 (24%)	0.191	0.909
Class II	57 (57%)	54 (54%)		
Class III	21 (21%)	22 (22%)		

Data are reported as number (%) or mean±SD. Diagnostic criteria for the three classifications of hypertension: Class I: 140-159/90-99 mmHg; Class II: 160-179/100-109 mmHg; Class III: ≥180/≥110 mmHg.

### Blood pressure control rate

Following the intervention, the proportion of patients achieving target blood pressure was markedly higher in the intervention group (98%) in contrast to the control group (84%) (χ^2^=11.966, P<0.05).

### SBP and DBP

Prior to the intervention, SBP and DBP were comparable between the two groups (P>0.05). Following the intervention, both groups exhibited substantial decreases in SBP and DBP compared to their pre-intervention levels (P<0.05). Moreover, post-intervention blood pressure values were lower in the intervention group versus the control group (*t*=20.640 and 13.033, respectively; P<0.05; Table 2).

**Table 2:** Comparison of blood pressure levels before and after intervention between the two groups.

Group	Intervention group (n=100)	Control group (n=100)	*t*	P
Systolic blood pressure				
Pre-intervention	152.13±5.08	152.40±4.69	0.387	0.699
Post-intervention	104.12±3.89*	115.30±3.77*	20.640	<0.001
Diastolic blood pressure				
Pre-intervention	101.53±3.82	101.55±3.70	0.034	0.973
Post-intervention	73.56±3.83*	81.08±4.31*	13.033	<0.001

Data are reported as mean±SD (mmHg). *P<0.05, compared to the same group before intervention.

### Medication adherence

Medication adherence was observed in 98 patients (98%) in the intervention group compared to 85 patients (85%) in the control group. The overall adherence rate was significantly higher in the intervention group (χ^2^=10.865, P<0.05; Table 3).

**Table 3:** Comparison of medication adherence between the two groups.

Group	n	Complete adherence	Partial adherence	Non-adherence	Overall compliance rate
Intervention group	100	67 (67%)	31 (31%)	2 (2%)	98 (98%)
Control group	100	56 (56%)	29 (29%)	15 (15%)	85 (85%)
χ^2^					10.865
P					0.001

Data are reported as number and percent.

### Self-management ability

Prior to the intervention, there were no differences in the HPSMBRS scale scores for dietary management, medication management, emotional management, exercise management, disease monitoring, and work-rest balance management between the two groups (P>0.05). Following the intervention, both groups demonstrated notable improvements in HPSMBRS scale scores compared to their pre-intervention scores (P<0.05), with the intervention group achieving higher post-intervention scores across all domains (P<0.05; Table 4).

**Table 4:** Comparison of the HPSMBRS scores between the two groups.

Group	Intervention group (n=100)	Control group (n=100)	z	P
Dietary management				
Pre-intervention	25.00 (24.00, 26.00)	25.00 (24.00, 27.00)	1.408	0.159
Post-intervention	41.00 (40.00, 42.00)*	32.00 (31.00, 34.00)*	12.230	<0.001
Medication management				
Pre-intervention	8.00 (6.50, 10.00)	8.00 (7.00, 9.00)	1.320	0.187
Post-intervention	16.00 (14.00, 18.00)*	13.00 (11.00, 16.00)*	5.710	<0.001
Emotional management				
Pre-intervention	13.00 (11.00, 14.00)	13.00 (12.00, 14.00)	0.779	0.436
Post-intervention	29.00 (27.00, 30.50)*	22.00 (21.00, 24.00)*	12.044	<0.001
Exercise management				
Pre-intervention	6.00 (5.00, 7.00)	6.00 (4.00, 7.00)	0.130	0.897
Post-intervention	13.00 (12.00, 14.00)*	9.00 (8.00, 10.00)*	10.487	<0.001
Disease monitoring				
Pre-intervention	7.00 (6.00, 8.00)	8.00 (6.00, 8.50)	1.469	0.142
Post-intervention	15.00 (13.00, 16.00)*	11.00 (10.00, 12.00)*	9.711	<0.001
Work-rest balance management				
Pre-intervention	8.00 (7.00, 10.00)	8.00 (6.50, 9.00)	0.184	0.854
Post-intervention	20.00 (19.00, 20.00)*	15.00 (13.00, 16.00)*	11.771	<0.001

Data are reported as median (P25, P75). *P<0.05, compared to the same group before intervention. HPSMBRS: Hypertension Patient Self-Management Behavior Rating Scale.

### Health behaviors

Baseline HPLP-II scores for health responsibility, physical exercise, nutrition, and stress management did not differ significantly between groups (P>0.05). Following the intervention, both groups experienced noticeable increases in HPLP-II scale scores compared to their pre-intervention scores (P<0.05), with the intervention group demonstrating superior performance across all assessed domains (P<0.05; Table 5).

**Table 5:** Comparison of the HPLP-II scale scores between the two groups.

Group	Intervention group (n=100)	Control group (n=100)	*t*	P
Health responsibility				
Pre-intervention	18.00 (15.50, 21.00)	18.00 (17.00, 20.50)	0.665	0.506
Post-intervention	29.00 (28.00, 31.00)*	25.00 (23.00, 28.00)*	7.997	<0.001
Physical exercise				
Pre-intervention	18.00 (16.00, 21.00)	19.00 (16.00, 21.00)	0.772	0.440
Post-intervention	29.00 (27.00, 31.50)*	24.00 (23.00, 27.00)*	8.510	<0.001
Nutrition				
Pre-intervention	18.00 (15.50, 20.00)	18.00 (15.00, 20.00)	0.416	0.677
Post-intervention	29.00 (27.00, 31.00)*	25.00 (23.00, 27.00)*	8.209	<0.001
Stress management				
Pre-intervention	18.00 (15.00, 19.00)	17.00 (15.50, 19.00)	1.305	0.192
Post-intervention	29.00 (27.00, 31.00)*	25.00 (23.00, 27.00)*	7.542	<0.001

Data are reported as median (P25, P75). *P<0.05, compared to the same group before intervention. HPLP-II: Health-Promoting Lifestyle Profile II.

### Quality of life

Prior to the intervention, GQOLI-74 scores across social function, psychological function, physical function, and material living conditions were comparable between groups (P>0.05). Both groups presented remarkable enhancements in GQOLI-74 scale scores compared to their pre-intervention scores post-intervention (P<0.05), with the intervention group showing consistently higher scores (P<0.05; Table 6).

**Table 6:** Comparison of the GQOLI-74 scale scores between the two groups.

Group	Intervention group (n=100)	Control group (n=100)	*t*	P
Social function				
Pre-intervention	56.44±7.37	56.14±6.84	0.298	0.766
Post-intervention	76.77±6.09*	69.65±5.42*	8.733	<0.001
Psychological function				
Pre-intervention	55.88±7.73	55.75±6.28	0.131	0.896
Post-intervention	80.51±5.47*	73.39±5.68*	9.030	<0.001
Physical function				
Pre-intervention	54.42±5.80	55.29±5.54	1.085	0.279
Post-intervention	79.68±6.10*	69.49±6.81*	11.145	<0.001
Material living conditions				
Pre-intervention	55.60±5.17	56.66±4.90	1.487	0.139
Post-intervention	76.19±5.47*	69.50±4.81*	9.181	<0.001

Data are reported as mean±SD. *P<0.05, compared to the same group before intervention. GQOLI-74: Generic Quality of Life Inventory-74.

### Patient satisfaction

Overall satisfaction with nursing care was reported by 97 patients (97%) in the intervention group and 83 patients (83%) in the control group. The intervention group demonstrated a notably higher satisfaction rate (χ^2^=10.889, P<0.05; Table 7).

**Table 7:** Comparison of nursing satisfaction between the two groups.

Group	n	Very satisfied	Satisfied	Dissatisfied	Total satisfaction rate
Intervention group	100	66 (66%)	31 (31%)	3 (3%)	97 (97%)
Control group	100	61 (61%)	22 (22%)	17 (17%)	83 (83%)
χ^2^					10.889
P					0.001

Data are reported as number and percent.

## Discussion

Hypertension remains a major contributor to global morbidity and mortality, primarily through its association with cardiovascular, cerebrovascular, and chronic kidney diseases, and continues to impose a substantial economic burden worldwide (16,17). Although advances in antihypertensive therapy and the implementation of evidence-based guidelines have improved blood pressure control, long-term hypertension management still largely depends on patients' sustained self-care behaviors and engagement in treatment (18,19). In this context, the present study elucidated that integrating information-based health management with the KAP health education model yields superior outcomes compared with conventional care across multiple clinical and behavioral domains.

The markedly higher blood pressure control rate observed in the intervention group highlights the effectiveness of this structured, behavior-oriented strategy. Digital platforms such as WeChat enabled continuous delivery of standardized health information and interactive guidance regarding medication use, diet, physical activity, and stress management. This finding is consistent with recent systematic reviews and randomized studies demonstrating that digital health-supported interventions significantly improve blood pressure control rates and reduce systolic blood pressure compared with usual care, particularly when combined with structured behavioral education (5,20). Importantly, the KAP framework ensured that information provision was systematically translated into attitudinal adjustment and behavioral practice, thereby enhancing blood pressure regulation beyond pharmacotherapy alone. Insufficient disease-related knowledge remains a common barrier to effective hypertension control and is closely associated with poor adherence and increased risk of complications (21). In the present study, the combined intervention addressed this gap through repeated, targeted education embedded within a structured KAP framework. Similar improvements in blood pressure outcomes have been reported in recent WeChat-based and mobile health-assisted hypertension management programs, highlighting the value of theory-driven education delivered through digital platforms (22). Rather than relying solely on increased healthcare contact, the intervention emphasized consistency, behavioral reinforcement, and patient engagement, which may partly explain its sustained effectiveness.

Medication adherence also improved under the combined intervention. By emphasizing the progression from knowledge acquisition to attitude formation and practical implementation, the KAP model helped mitigate common cognitive and behavioral barriers to long-term medication use. Recent randomized controlled trials have shown that mobile health interventions incorporating reminders, education, and interactive feedback can significantly enhance medication adherence and subsequently improve blood pressure outcomes (23). Consistent with prior evidence, adherence behaviors are influenced by professional guidance, social norms, and structured health education (24). The incorporation of digital group-based communication further strengthened peer support and accountability, contributing to more consistent medication-taking behaviors. Beyond adherence, patients receiving the combined intervention demonstrated superior self-management abilities and health-promoting behaviors, including dietary regulation, physical activity, disease monitoring, and stress management. Digital tracking and feedback mechanisms supported patients in reviewing their daily practices, thereby reinforcing self-efficacy and sustained engagement. These findings align with recent evidence indicating that mobile health applications can effectively promote self-management behaviors and improve long-term control of chronic conditions such as hypertension (25,26).

Improvements in quality of life further reflect the holistic benefits of the integrated intervention. Enhanced disease awareness, improved symptom control, and greater confidence in daily self-management likely contributed to gains across physical, psychological, and social domains. Recent research has similarly reported that digital health-supported hypertension management programs are associated with significant improvements in patient-reported outcomes and quality of life (26). The KAP model emphasizes aligning patient attitudes with evidence-based practices, which may promote long-term lifestyle modification and improved well-being (27,28). Increased disease insight may also strengthen patients' motivation to actively participate in health information seeking and self-care activities, thereby supporting sustained improvements in quality of life (13). Higher patient satisfaction observed in the intervention group highlights the value of patient-centered, technology-enabled care. The convenience of digital platforms for communication, education, and follow-up likely enhanced patients' perceptions of accessibility and responsiveness. Previous research has demonstrated that integrating health information technology with structured educational models can improve patient satisfaction while maintaining acceptable or reduced healthcare costs (29,30).

Our study supports the integration of digital health tools with practice-based educational frameworks such as the KAP model in hypertension management programs. By enhancing patient engagement and daily self-management practices, such interventions may improve population-level cardiovascular outcomes while promoting efficient use of healthcare resources. Nevertheless, the relatively short follow-up period and single-center design limit the assessment of long-term sustainability and generalizability. Future multicenter studies with extended follow-up and cost-effectiveness analyses are warranted.

## Data Availability

All data generated or analyzed during this study are included in this published article.
